# Results from the Survey of Antibiotic Resistance (SOAR) 2018–21 in Türkiye: data based on CLSI, EUCAST (dose-specific) and pharmacokinetic/pharmacodynamic (PK/PD) breakpoints

**DOI:** 10.1093/jac/dkaf289

**Published:** 2025-11-24

**Authors:** Didem Torumkuney, Nergis Keles, Ufuk Hasdemir, Gülşen Hazırolan, Sohret Aydemir, Zerrin Aktas, Oral Oncul, Ian Morrissey, Anand Manoharan

**Affiliations:** Infectious Diseases Research Unit, GSK, London, UK; Infectious Diseases Medical & Scientific Affairs, GSK, Istanbul, Türkiye; Department of Medical Microbiology, Marmara University School of Medicine, Pendik Training and Research Hospital, Istanbul, Türkiye; Department of Medical Microbiology, Hacettepe University Faculty of Medicine, Ankara, Türkiye; Department of Clinical Microbiology, Faculty of Medicine, Ege University, Izmir, Türkiye; Department of Clinical Microbiology, Istanbul Faculty of Medicine, Istanbul University, Istanbul, Türkiye; Department of Infectious Diseases and Clinical Microbiology, Istanbul Faculty of Medicine, Istanbul University, Istanbul, Türkiye; Antimicrobial Focus Ltd., Sawbridgeworth, UK; Infectious Diseases Medical & Scientific Affairs, GSK, Mumbai, India

## Abstract

**Objectives:**

To determine the antibiotic susceptibility of *Streptococcus pneumoniae* and *Haemophilus influenzae* from community-acquired respiratory tract infections from four hospitals in Türkiye in 2018–21.

**Methods:**

MICs were determined by CLSI methodology, and susceptibility was interpreted using CLSI, EUCAST and pharmacokinetic/pharmacodynamic (PK/PD) breakpoints.

**Results:**

*S. pneumoniae* (*n* = 142) and *H. influenzae* (*n* = 315) isolates were collected. Penicillin susceptibility in pneumococci was 35.9% by CLSI oral/EUCAST low-dose breakpoints and 82.4% by EUCAST high-dose/CLSI intravenous administration. Similar susceptibility (78.2%–82.4%, CLSI) was observed for ceftriaxone, amoxicillin/clavulanic acid, amoxicillin and cefotaxime. Other cephalosporins, tetracyclines, macrolides and trimethoprim/sulfamethoxazole were poorly active (42.3%–59.2%); fluoroquinolones (≥97.9%) were most active. Susceptibility by EUCAST was lower for high-dose amoxicillin (64.8%), high-dose amoxicillin/clavulanic acid (59.2%) and cefaclor (0%), but higher for high-dose ceftriaxone (99.3%). High-dose PK/PD breakpoints for amoxicillin and amoxicillin/clavulanic acid increased susceptibility (87.3% and 88.0%). Most *H. influenzae* were β-lactamase negative (*n* = 294, 93.3%); 26 (8.3%) were β-lactamase-negative ampicillin-resistant by EUCAST breakpoints and eight (2.5%) by CLSI breakpoints. Antibiotic susceptibility was ≥91.8% (CLSI) except ampicillin (85.4%) and trimethoprim/sulfamethoxazole (64.1%). EUCAST or PK/PD breakpoint susceptibility was similar, except for oral cefuroxime (0% EUCAST, 71.7% PK/PD and 99.4% CLSI) and macrolides (no EUCAST breakpoint, 1.0%–2.5% PK/PD and 96.5%–99.1% CLSI).

**Conclusions:**

Susceptibility in *S. pneumoniae* was <60% except for fluoroquinolones, amoxicillin and amoxicillin/clavulanic acid, cefotaxime and ceftriaxone. *H. influenzae* susceptibility to most antibiotics was >90% except for trimethoprim/sulfamethoxazole and ampicillin. These data are similar to recent surveillance of antibiotic resistance data in Türkiye.

## Introduction

Community-acquired respiratory tract infections (CA-RTIs) are an important global health issue that can result in hospitalization if treated inappropriately, or if occurring in patients with comorbidities; a third of patients with community-acquired pneumonia die within 12 months after being discharged from hospital.^1^ However, mortality rate may have been influenced by comorbidities, age and other risk factors.^1^ Treatment of CA-RTIs relies on empirical antibiotic therapy, as recommended by national and international guidelines.^2^ Reviews of antibiotic consumption in Eastern Europe in 2011 and 2015 showed that Türkiye ranked highest in consumption of antibiotics for systemic use.^3^ Antibiotic consumption data have been shown to vary by province or region in Türkiye; this is attributed to differing climate and seasonal weather variability and level of access to healthcare.^4^ Inappropriate antibiotic use is also associated with resistance development,^5^ which is high in Türkiye, with 35%–55% of patients discontinuing antibiotic medication before completing the course and around 30% of patients reported to use antibiotics without a prescription.^6,7^ Although over-the-counter sales of antibiotics have been prohibited in Türkiye since 2015, a web-based survey among 945 Turkish-speaking respondents reported that the second most common way of obtaining antibiotics was from leftovers (20.3%).^7^

The major bacteria associated with CA-RTIs are *S. pneumoniae* and *H. influenzae*;^8,9^ both pathogens have shown increasing resistance to first-line antibiotics such as penicillin and ampicillin.^10,11^ Up-to-date surveillance data are essential to guide local antibiotic policies, as rates of resistance vary over time and from country to country.^12^

An international antibiotic resistance surveillance study, the Survey of Antibiotic Resistance (SOAR), focuses on key respiratory pathogens that cause community-acquired infections. The SOAR has been running since 2002 in the Middle East, Africa, Latin America, Asia-Pacific, Europe and the Commonwealth of Independent States countries.^13^ For this study, analysis of the recent SOAR data from hospitals in Türkiye has been conducted to depict the current state of antibiotic susceptibility of *S. pneumoniae* and *H. influenzae* associated with CA-RTIs.

## Materials and methods

### Ethics

SOAR studies are not human subject studies. During the study, only microorganisms were examined. Permission for the use of patient information in this study was obtained from the ethics committee for Istanbul University, Istanbul Faculty of Medicine.

### Collaborating centres

Isolates were collected between 2018 and 2021 from the following four centres across Izmir and Istanbul: Ege University Faculty of Medicine; Hacettepe University Faculty of Medicine; Istanbul University, Istanbul Faculty of Medicine; and Marmara University Pendik Training and Research Hospital.

### Clinical isolates

Isolates of *H. influenzae* and *S. pneumoniae* from CA-RTIs (isolated within 48 h of hospitalization) were sent to be sub-cultured and re-identified at a central laboratory (IHMA Europe, Monthey, Switzerland). MALDI-TOF MS methodology was used to reidentify *H. influenzae;* β-lactamase production for each *H. influenzae* isolate was determined by a chromogenic cephalosporin (nitrocefin) disc method. *S. pneumoniae* identity was confirmed by optochin susceptibility and bile solubility. Duplicate isolates from the same patient were not accepted.

### Susceptibility testing

Antibiotic susceptibility was evaluated in isolates using broth microdilution methodology recommended by CLSI.^14^ Both respiratory pathogens were tested against amoxicillin, amoxicillin/clavulanic acid (2:1 ratio as per CLSI guidelines^14,15^), amoxicillin/clavulanic acid (fixed clavulanic acid at 2 mg/L as per EUCAST guidelines^16^), azithromycin, cefaclor, cefdinir, cefixime, cefotaxime, cefpodoxime, ceftibuten, ceftriaxone, cefuroxime, clarithromycin, levofloxacin, moxifloxacin, tetracycline and trimethoprim/sulfamethoxazole (1:19 ratio). In addition, doxycycline, erythromycin and penicillin were tested against *S. pneumoniae* only, and ampicillin was tested against *H. influenzae* only. Susceptibility to the study drugs was calculated based on CLSI breakpoints, EUCAST (dose-specific) breakpoints and pharmacokinetic/pharmacodynamic (PK/PD) breakpoints,^15–17^ given in Tables 1–3. Susceptibility using EUCAST criteria was calculated by combining percentage susceptible and susceptible, increased exposure into the susceptible category as well as dose-dependent PK/PD breakpoints,^16,17^ to fully assess antibiotics where high-dose therapies are available. The antibiotics with high-dose availability assessed in this way included: amoxicillin (0.75–1 g oral, 3×daily), amoxicillin/clavulanic acid (0.875 g amoxicillin/0.125 g clavulanic acid oral, 3×daily), ampicillin (2 g intravenous [IV], 4×daily), penicillin (2.4 g IV, 2 MU 4–6×daily), ceftriaxone (2 g IV, 2×daily), clarithromycin (0.5 g oral, 2×daily), erythromycin (1 g oral or IV, 4×daily), levofloxacin (0.75 g oral 2×daily, or 0.4 g IV 3×daily) and trimethoprim/sulfamethoxazole (0.24 g trimethoprim/1.2 g sulfamethoxazole oral or IV, 2×daily).^16^

**Table 1.** CLSI MIC breakpoints (mg/L) used for *S. pneumoniae* and *H. influenzae* isolates

	*S. pneumoniae*			*H. influenzae*		
Antimicrobial	S	I	R	S	I	R
Amoxicillin	≤2	4	≥8	—	—	—
Amoxicillin/clavulanic acid (2:1)^a^	≤2	4	≥8	≤2	4	≥8
Ampicillin	NT	NT	NT	≤1	2	≥4
Azithromycin	≤0.5	1	≥2	≤4	—	—
Cefaclor	≤1	2	≥4	≤8	16	≥32
Cefdinir	≤0.5	1	≥2	≤1	—	—
Cefixime	—	—	—	≤1	—	—
Cefotaxime (non-meningitis)	≤1	2	≥4	≤2	—	—
Cefpodoxime	≤0.5	1	≥2	≤2	—	—
Ceftibuten	—	—	—	≤2	—	—
Ceftriaxone (non-meningitis)	≤1	2	≥4	≤2	—	—
Cefuroxime^b^	≤1	2	≥4	≤4	8	≥16
Clarithromycin	≤0.25	0.5	≥1	≤8	16	≥32
Doxycycline	≤0.25	0.5	≥1	NT	NT	NT
Erythromycin	≤0.25	0.5	≥1	NT	NT	NT
Levofloxacin	≤2	4	≥8	≤2	—	—
Moxifloxacin	≤1	2	≥4	≤1	—	—
Penicillin (2.4 g 2 MU × 4–6 IV)	≤2	4	≥8	NT	NT	NT
Penicillin (oral)	≤0.06	0.12–1	≥2	NT	NT	NT
Tetracycline	≤1	2	≥4	≤2	4	≥8
Trimethoprim/sulfamethoxazole^c^	≤0.5	1–2	≥4	≤0.5	1–2	≥4

—, not applicable; I, intermediate; NT, not tested; R, resistant; S, susceptible.

^a^Amoxicillin/clavulanic acid was tested at a 2:1 amoxicillin-to-clavulanic acid ratio; breakpoints are expressed as the amoxicillin component.

^b^Breakpoints used are for cefuroxime axetil (oral).

^c^Trimethoprim/sulfamethoxazole was tested at a 1:19 trimethoprim-to-sulfamethoxazole ratio; breakpoints are expressed as the trimethoprim component.

**Table 2.** EUCAST (dose-specific) MIC breakpoints (mg/L) used for *S. pneumoniae* and *H. influenzae* isolates

	*S. pneumoniae*		*H. influenzae*	
Antimicrobial^a^	S	R	S	R
Amoxicillin (0.5 g × 3 oral)	≤0.5	>1	≤0.001	>2
Amoxicillin (0.75–1 g × 3 oral)	≤1	>1	≤2	>2
Amoxicillin/clavulanic acid (0.5 g/0.125 g × 3 oral)^b^	≤0.5	>1	≤0.001	>2
Amoxicillin/clavulanic acid (0.875 g/0.125 g × 3 oral)^b^	≤1	>1	≤2	>2
Ampicillin (2 g × 3 IV)	NT	NT	≤1	>1
Ampicillin (2 g × 4 IV)	NT	NT	≤1	>1
Azithromycin	≤0.25	>0.5	—	—
Cefaclor	≤0.001	>0.5	—	—
Cefdinir	—	—	—	—
Cefixime	—	—	≤0.12	>0.12
Cefotaxime	≤0.5	>2	≤0.12	>0.12
Cefpodoxime	≤0.25	>0.5	≤0.25	>0.25
Ceftibuten	—	—	≤1	>1
Ceftriaxone (1 g × 1 IV)	≤0.5	>2	≤0.12	>0.12
Ceftriaxone (2 g × 2 IV)	≤2	>2	≤0.12	>0.12
Cefuroxime^c^	≤0.25	>0.5	≤0.001	>1
Clarithromycin (0.25 g × 2 oral)	≤0.25	>0.5	—	—
Clarithromycin (0.5 g × 2 oral)	≤0.5	>0.5	—	—
Doxycycline	≤1	>2	NT	NT
Erythromycin (0.5 g × 2–4 oral or 0.5 g × 2–4 IV)	≤0.25	>0.5	NT	NT
Erythromycin (1 g × 4 oral or 1 g × 4 IV)	≤0.5	>0.5	NT	NT
Levofloxacin (0.5 g × 2 oral or 0.4 g × 2 IV)	≤0.001	>2	≤0.06	>0.06
Levofloxacin (0.75 g × 2 oral or 0.4 g × 3 IV)	≤2	>2	≤0.06	>0.06
Moxifloxacin	≤0.5	>0.5	≤0.12	>0.12
Penicillin (0.6 g 1 MU × 4 IV)	≤0.06	>2	NT	NT
Penicillin (2.4 g 2 MU × 4–6 IV)	≤2	>2	NT	NT
Tetracycline	≤1	>2	≤2	>2
Trimethoprim/sulfamethoxazole (0.16 g/0.8 g × 2 oral or IV)^d^	≤1	>2	≤0.5	>1
Trimethoprim/sulfamethoxazole (0.24 g/1.2 g × 2 oral or IV)^d^	≤2	>2	≤1	>1

—, not applicable; NT, not tested; R, resistant; S, susceptible.

^a^Where available, susceptibility was assessed using EUCAST higher dosage breakpoints.

^b^Amoxicillin/clavulanic acid was tested at a fixed concentration of 2 mg/L; breakpoints are expressed as the amoxicillin component.

^c^Breakpoints used are for cefuroxime axetil (oral).

^d^Trimethoprim/sulfamethoxazole was tested at a 1:19 trimethoprim-to-sulfamethoxazole ratio; breakpoints are expressed as the trimethoprim component.

**Table 3.** PK/PD MIC breakpoints (mg/L) used for *S. pneumoniae* and *H. influenzae* isolates

	*S. pneumoniae* and *H. influenzae*
Antimicrobial	S only
Amoxicillin (1.5 g/day)^a^	≤2
Amoxicillin (4 g/day)^b^	≤4
Amoxicillin/clavulanic acid^a^ (1.75 g/0.25 g/day adults; 45 mg/6.4 mg/kg/day children)	≤2
Amoxicillin/clavulanic acid^b^ (4 g/0.25 g/day adults; 90 mg/6.4 mg/kg/day children)	≤4
Ampicillin	—
Penicillin	—
Cefaclor	≤0.5
Cefdinir	≤0.25
Cefditoren	—
Cefixime	≤1
Cefpodoxime	≤0.5
Ceftriaxone	≤1
Cefuroxime^c^	≤1
Azithromycin	≤0.12
Clarithromycin	≤0.25
Erythromycin	≤0.25
Levofloxacin	≤2
Moxifloxacin	≤1
Trimethoprim/sulfamethoxazole^d^	≤0.5

—, not applicable; PK/PD, pharmacokinetic/pharmacodynamic; S, susceptible.

^a^Amoxicillin/clavulanic acid for low dose in adults/children.

^b^Amoxicillin/clavulanic acid for high dose in adults/children.

^c^Breakpoints used are for cefuroxime axetil (oral).

^d^Trimethoprim/sulfamethoxazole was tested at a 1:19 trimethoprim-to-sulfamethoxazole ratio; breakpoints are expressed as the trimethoprim component.

### Quality control and data analysis

Quality control strains *S. pneumoniae* ATCC 49619, *H. influenzae* ATCC 49247, *H. influenzae* ATCC 49766 and *E. coli* ATCC 32518 were included on each day of testing. The results of susceptibility testing were only accepted if the results of the quality control strains were within the published acceptable range. Across penicillin susceptibility (*S. pneumoniae* only), differences in susceptibility (using CLSI criteria) were assessed for statistical significance with Fisher’s exact test using XLSTAT version 2023.1.1.1399. A *P* value <0.05 was considered statistically significant. A similar statistical analysis was performed to compare the susceptibility of isolates from this study period (2018–21) with SOAR data from Türkiye 2015–17 (using CLSI criteria).^18^

## Results

### *S. pneumoniae* isolates

A total of 142 *S. pneumoniae* isolates were collected from four centres in Türkiye between 2018 and 2021. Most isolates came from sputum (*n* = 84, 59.2%), with the remainder from endotracheal aspirate (*n* = 26, 18.3%), blood (*n* = 10, 7.0%), bronchoalveolar lavage (*n* = 9, 6.3%) and unidentified specimens (*n* = 13, 9.2%). Most isolates (*n* = 83, 58.5%) came from adolescent and adult patients (aged 13–64 years), 36 (25.4%) isolates were from elderly patients (aged ≥65 years) and 23 (16.2%) isolates from paediatric patients (aged ≤12 years).

**Figure 1.**
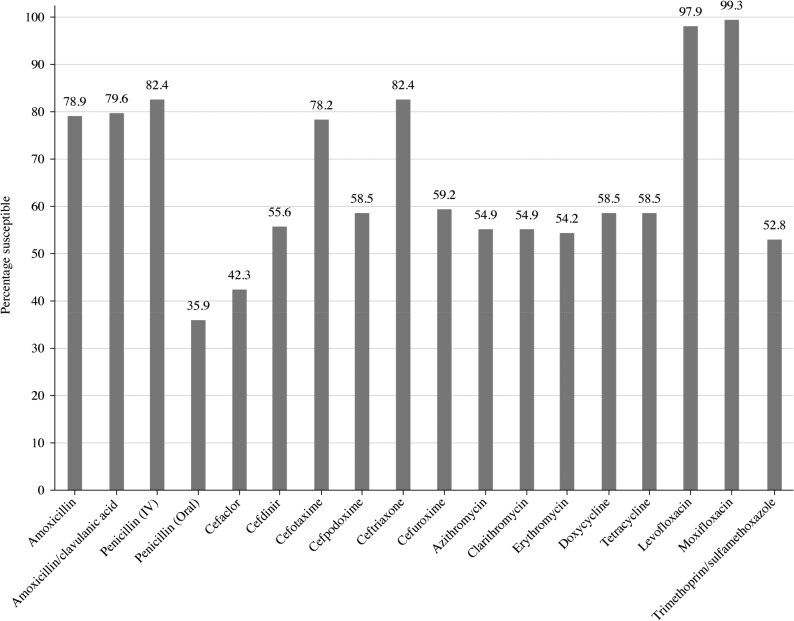
Antibiotic susceptibility rates of *S. pneumoniae* isolates (*n* = 142) from Türkiye based on CLSI breakpoints.

**Figure 2.**
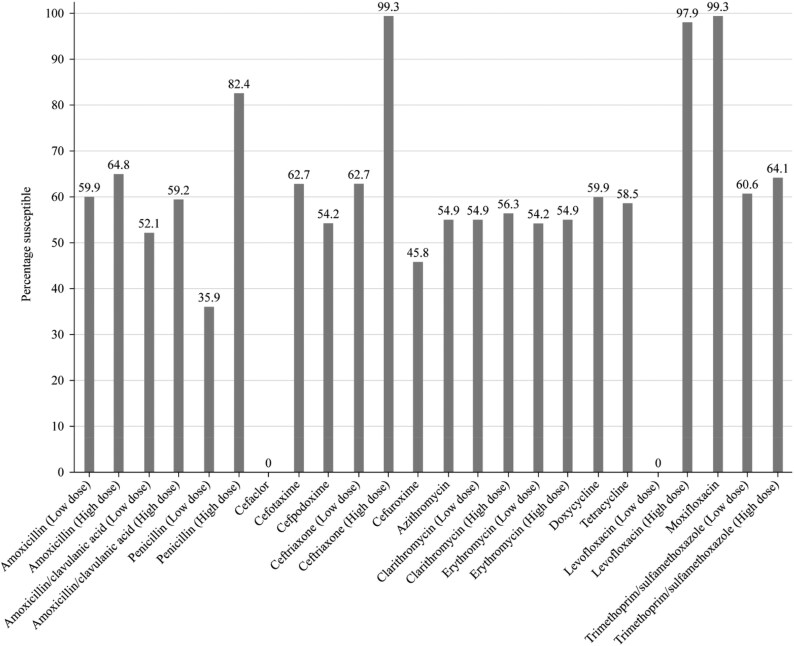
Antibiotic susceptibility rates of *S. pneumoniae* isolates (*n* = 142) from Türkiye based on EUCAST (dose-specific) breakpoints.

Summary MIC, susceptibility and MIC distribution data for all 142 *S. pneumoniae* isolates are given in Tables 4–6 and S1 (available as Supplementary data at *JAC* Online) and shown in Figures 1 and 2.

**Table 4.** MIC and susceptibility data for *S. pneumoniae* isolates (*n* = 142) from Türkiye using CLSI breakpoints

	MIC (mg/L)			CLSI susceptibility		
Antimicrobial	Range	50%	90%	%S	%I	%R
Amoxicillin	≤0.008–>8	0.25	8	78.9	8.5	12.7
Amoxicillin/clavulanic acid (2:1)	≤0.008–>8	0.25	8	79.6	8.5	12.0
Penicillin (2.4 g 2 MU × 4–6 IV)	≤0.008–8	0.25	4	82.4	16.9	0.7
Penicillin (oral)	≤0.008–8	0.25	4	35.9	32.4	31.7
Cefaclor	0.25–>4	4	>4	42.3	5.6	52.1
Cefdinir	0.03–>8	0.5	>8	55.6	3.5	40.8
Cefixime	≤0.25–>16	2	>16	—	—	—
Cefotaxime	≤0.008–4	0.12	2	78.2	19.0	2.8
Cefpodoxime	≤0.015–>4	0.25	>4	58.5	4.9	36.6
Ceftibuten	2–>16	>16	>16	—	—	—
Ceftriaxone	≤0.008–4	0.25	2	82.4	16.9	0.7
Cefuroxime	≤0.008–>8	0.5	>8	59.2	4.9	35.9
Azithromycin	≤0.015–>16	0.06	>16	54.9	0.7	44.4
Clarithromycin	≤0.015–>16	0.03	>16	54.9	1.4	43.7
Erythromycin	≤0.015–>16	0.03	>16	54.2	0.7	45.1
Doxycycline	0.03–>4	0.06	>4	58.5	0.7	40.8
Tetracycline	0.06–>4	0.25	>4	58.5	0.7	40.8
Levofloxacin	0.5–>8	1	1	97.9	1.4	0.7
Moxifloxacin	≤0.03–4	0.12	0.12	99.3	0	0.7
Trimethoprim/sulfamethoxazole	≤0.06–>8	0.5	>8	52.8	11.3	35.9

—, not applicable; I, intermediate; R, resistant; S, susceptible.

**Table 5.** MIC and susceptibility data for *S. pneumoniae* isolates (*n* = 142) from Türkiye using EUCAST (dose-specific) breakpoints

	MIC (mg/L)			EUCAST susceptibility		
Antimicrobial	Range	50%	90%	%S	%I	%R
Amoxicillin (0.5 g × 3 oral)	≤0.008–>8	0.25	8	59.9	4.9	35.2
Amoxicillin (0.75–1 g × 3 oral)	≤0.008–>8	0.25	8	64.8	—	35.2
Amoxicillin/clavulanic acid (0.5 g/0.125 g × 3 oral)	0.015–>8	0.5	>8	52.1	7.0	40.9
Amoxicillin/clavulanic acid (0.875 g/0.125 g × 3 oral)	0.015–>8	0.5	>8	59.2	—	40.9
Penicillin (0.6 g 1 MU × 4 IV)	≤0.008–8	0.25	4	35.9	46.5	17.6
Penicillin (2.4 g 2 MU × 4–6 IV)	≤0.008–8	0.25	4	82.4	—	17.6
Cefaclor	0.25–>4	4	>4	0	34.5	65.5
Cefdinir	0.03–>8	0.5	>8	—	—	—
Cefixime	≤0.25–>16	2	>16	—	—	—
Cefotaxime	≤0.008–4	0.12	2	62.7	34.5	2.8
Cefpodoxime	≤0.015–>4	0.25	>4	54.2	4.2	41.5
Ceftibuten	2–>16	>16	>16	—	—	—
Ceftriaxone (1 g × 1 IV)	≤0.008–4	0.25	2	62.7	36.6	0.7
Ceftriaxone (2 g × 2 IV)	≤0.008–4	0.25	2	99.3	—	0.7
Cefuroxime	≤0.008–>8	0.5	>8	45.8	11.3	43.0
Azithromycin	≤0.015–>16	0.06	>16	54.9	0	45.1
Clarithromycin (0.25 g × 2 oral)	≤0.015–>16	0.03	>16	54.9	1.4	43.7
Clarithromycin (0.5 g × 2 oral)	≤0.015–>16	0.03	>16	56.3	—	43.7
Erythromycin (0.5 g × 2–4 oral or 0.5 g × 2–4 IV)	≤0.015–>16	0.03	>16	54.2	0.7	45.1
Erythromycin (1 g × 4 oral or 1 g × 4 IV)	≤0.015–>16	0.03	>16	54.9	—	45.1
Doxycycline	0.03–>4	0.06	>4	59.9	4.2	35.9
Tetracycline	0.06–>4	0.25	>4	58.5	0.7	40.8
Levofloxacin (0.5 g × 2 oral or 0.4 g × 2 IV)	0.5–>8	1	1	0	97.9	2.1
Levofloxacin (0.75 g × 2 oral or 0.4 g × 3 IV)	0.5–>8	1	1	97.9	—	2.1
Moxifloxacin	≤0.03–4	0.12	0.12	99.3	—	0.7
Trimethoprim/sulfamethoxazole (0.16 g/0.8 g × 2 oral or IV)	≤0.06–>8	0.5	>8	60.6	3.5	35.9
Trimethoprim/sulfamethoxazole (0.24 g/1.2 g × 2 oral or IV)	≤0.06–>8	0.5	>8	64.1	—	35.9

—, not applicable; I, susceptible, increased exposure; R, resistant; S, susceptible.

**Table 6.** Summary MIC and susceptibility data for *S. pneumoniae* (*n* = 142) from Türkiye using PK/PD breakpoints

	MIC (mg/L)			PK/PD susceptibility
Antimicrobial	Range	50%	90%	%S
Amoxicillin (1.5 g/day)	≤0.008–>8	0.25	8	78.9
Amoxicillin (4 g/day)	≤0.008–>8	0.25	8	87.3
Amoxicillin/clavulanic acid (1.75 g/0.25 g/day adults; 45 mg/6.4 mg/kg/day children)	≤0.008–>8	0.25	8	79.6
Amoxicillin/clavulanic acid (4 g/0.25 g/day adults; 90 mg/6.4 mg/kg/day children)	≤0.008–>8	0.25	8	88.0
Penicillin	≤0.008–8	0.25	4	—
Cefaclor	0.25–>4	4	>4	34.5
Cefdinir	0.03–>8	0.5	>8	43.7
Cefixime	≤0.25–>16	2	>16	43.0
Cefotaxime	≤0.008–4	0.12	2	—
Cefpodoxime	≤0.015–>4	0.25	>4	58.5
Ceftibuten	2–>16	>16	>16	—
Ceftriaxone	≤0.008–4	0.25	2	82.4
Cefuroxime	≤0.008–>8	0.5	>8	59.2
Azithromycin	≤0.015–>16	0.06	>16	54.2
Clarithromycin	≤0.015–>16	0.03	>16	54.9
Erythromycin	≤0.015–>16	0.03	>16	54.2
Doxycycline	0.03–>4	0.06	>4	58.5
Tetracycline	0.06–>4	0.25	>4	—
Levofloxacin	0.5–>8	1	1	97.9
Moxifloxacin	≤0.03–4	0.12	0.12	99.3
Trimethoprim/sulfamethoxazole	≤0.06–>8	0.5	>8	52.8

—, not applicable; PK/PD, pharmacokinetic/pharmacodynamic; S, susceptible.

### *S. pneumoniae* susceptibility

When CLSI oral or EUCAST low-dose IV breakpoints were applied, only 35.9% of the 142 *S. pneumoniae* isolates collected in Türkiye were penicillin-susceptible (PSSP). However, susceptibility to penicillin with EUCAST high-dose and CLSI IV breakpoints increased to 82.4%. According to CLSI breakpoints, amoxicillin, amoxicillin/clavulanic acid and the third-generation cephalosporins cefotaxime and ceftriaxone showed similar activity, with susceptibility ranging from 78.2% to 82.4%; the third-generation cephalosporin cefdinir was less active (55.6% susceptible). Cefaclor, cefpodoxime and cefuroxime were also poorly active according to CLSI breakpoints (42.3%–59.2% susceptible). Similar data were obtained using PK/PD breakpoints, except higher dosing breakpoints increased amoxicillin susceptibility to 87.3% and amoxicillin/clavulanic acid susceptibility to 88.0%. EUCAST breakpoints for amoxicillin and amoxicillin/clavulanic acid are more conservative, resulting in lower susceptibility than that obtained by CLSI or PK/PD breakpoints (52.1%–64.8%), even at higher dosing regimens. Cephalosporin susceptibility by EUCAST breakpoints indicated suboptimal activity (0% susceptible for cefaclor to 62.7% susceptible for cefotaxime), except for high-dose ceftriaxone (99.3% susceptible). Similarly, poor activity was observed for the macrolides (azithromycin, clarithromycin and erythromycin), tetracyclines (doxycycline and tetracycline) and trimethoprim/sulfamethoxazole by CLSI, EUCAST and PK/PD breakpoint interpretation (54.9%–64.1% susceptibility). Moxifloxacin susceptibility was 99.3% using each breakpoint; similarly, levofloxacin susceptibility was 97.9% by CLSI, PK/PD and EUCAST high dose (Tables 4–6 and Figures 1 and 2).

### Comparative susceptibility of *S. pneumoniae* by penicillin susceptibility

**Figure 3.**
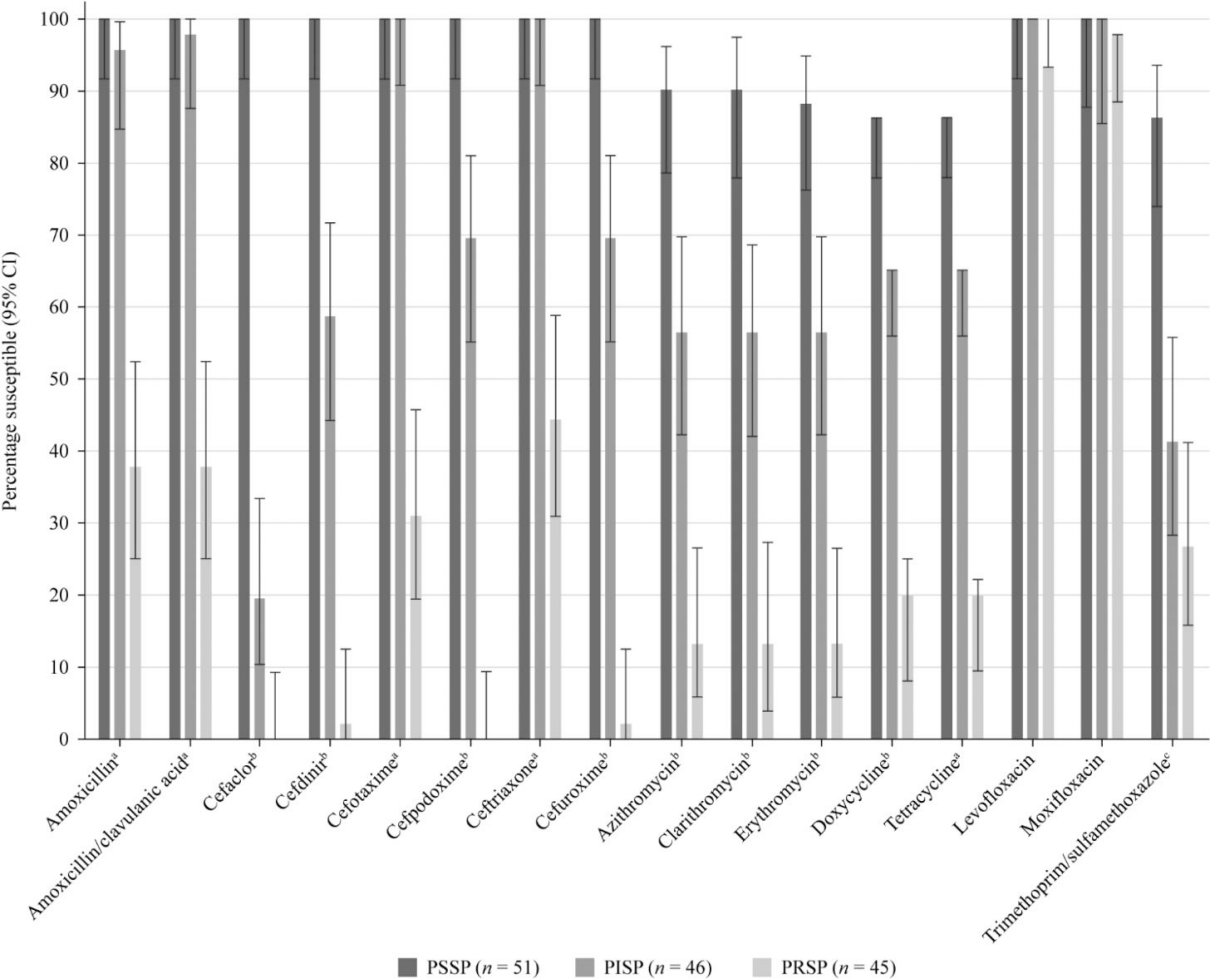
Susceptibility rates (with 95% CI) based on CLSI breakpoints for antibiotics against PSSP, PISP and PRSP from Türkiye. Penicillin susceptibility categories are based on oral penicillin CLSI breakpoints. ^a^Susceptibility was significantly higher among PSSP and PISP isolates than PRSP isolates (*P* < 0.0001). ^b^Susceptibility was significantly higher among PSSP than PISP isolates and among PISP than PRSP isolates (*P* < 0.003). ^c^Susceptibility was significantly higher among PSSP isolates than PISP and PRSP isolates (*P* < 0.0001). CI, confidence interval; PISP, penicillin-intermediate *S. pneumoniae*; PRSP, penicillin-resistant *S. pneumoniae;* PSSP, penicillin-susceptible *S. pneumoniae*.

Of the 142 *S. pneumoniae* isolates collected, 51 (35.9%) were PSSP, 46 (32.4%) were penicillin-intermediate (PISP) and 45 (31.7%) were penicillin-resistant (PRSP) according to CLSI oral breakpoints (Figure 3). All PSSP isolates were ≥86.3% susceptible to all antibiotics tested. PSSP isolates showed significantly higher (*P* < 0.0001) susceptibility rates than PRSP isolates for all antibiotics except the fluoroquinolones, which showed excellent activity irrespective of penicillin susceptibility. PSSP isolates also had significantly higher susceptibility than PISP isolates to cefaclor, cefdinir, cefpodoxime, cefuroxime, macrolides and tetracyclines. Susceptibility of PISP isolates to the remaining antibiotics was 95.7% (amoxicillin), 97.8% (amoxicillin/clavulanic acid) and 100% (cefotaxime, ceftriaxone and fluoroquinolones). Susceptibility rates of 0%–44.4% were observed against PRSP isolates for all antibiotics, except levofloxacin (93.3% susceptible) and moxifloxacin (97.8% susceptible).

### Comparative susceptibility of *S. pneumoniae* collected in 2015–17 and 2018–21

**Figure 4.**
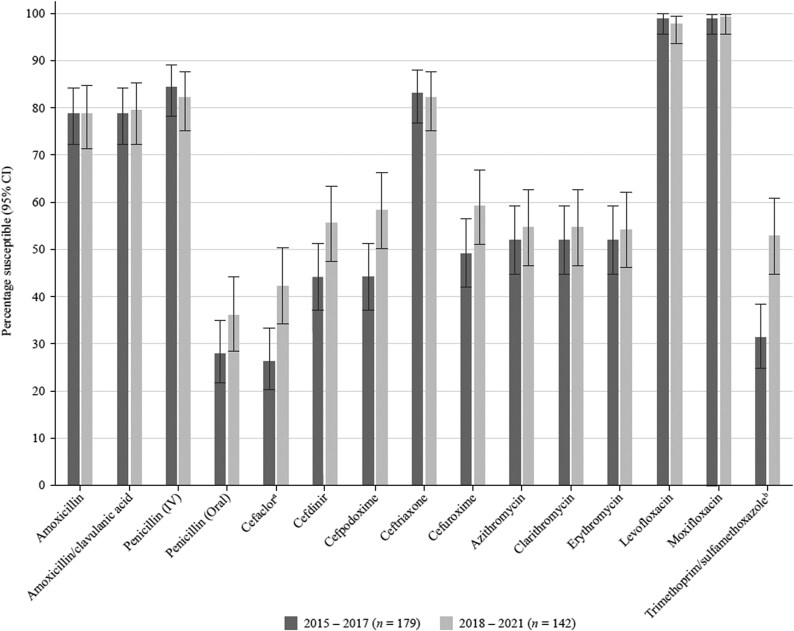
Comparison of antibiotic susceptibility rates of *S. pneumoniae* isolates from Türkiye collected in 2015–17 with isolates collected in 2018–21 (CLSI breakpoints). ^a^Susceptibility was significantly higher in 2018–21 than in 2015–17 (*P* = 0.003). ^b^Susceptibility was significantly higher in 2018–21 than in 2015–17 (*P* = 0.0001). CI, confidence interval.

Data have previously been published from the SOAR surveillance for the period 2015–17 and were compared for mutually tested antibiotics with the current study (2018–21) (Figure 4). There was no significant change in susceptibility except for an increased level of susceptibility to cefaclor (26.3% versus 42.3%) and trimethoprim/sulfamethoxazole (31.3% versus 52.8%). However, this improved activity would still be considered too low for therapeutic consideration.

### *H. influenzae* isolates

A total of 315 *H. influenzae* isolates were collected in Türkiye from 2018 to 2021. Most isolates originated from sputum (*n* = 254, 80.6%). The remaining isolates were from endotracheal aspirate (*n* = 30, 9.5%), bronchoalveolar lavage (*n* = 26, 8.3%), blood (*n* = 1, 0.3%) and unidentified specimens (*n* = 4, 1.3%). Just over half of the isolates (*n* = 177, 56.2%) came from adolescent and adult patients (aged 13–64 years), 85 (27.0%) isolates were from elderly patients (aged ≥65 years), and 53 (16.8%) isolates were from paediatric patients (aged ≤12 years).

**Figure 5.**
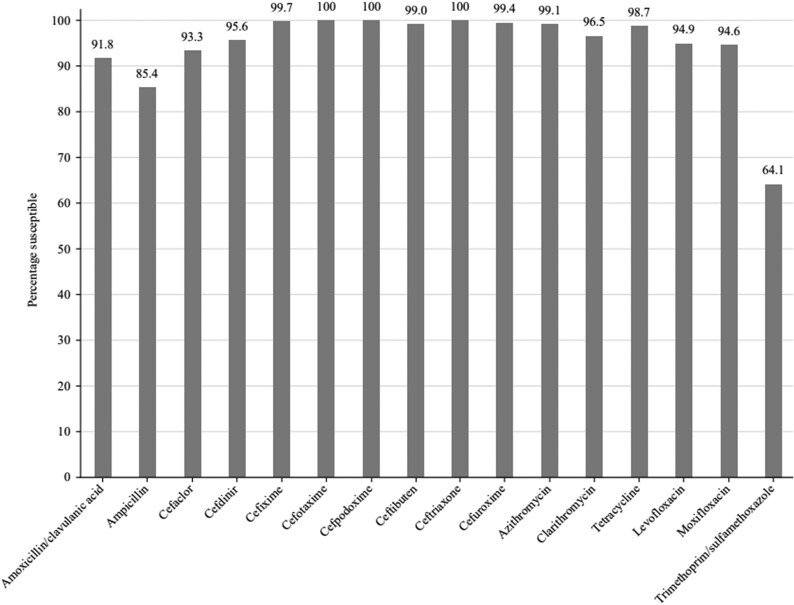
Antibiotic susceptibility rates of *H. influenzae* isolates (*n* = 315) from Türkiye based on CLSI breakpoints.

**Figure 6.**
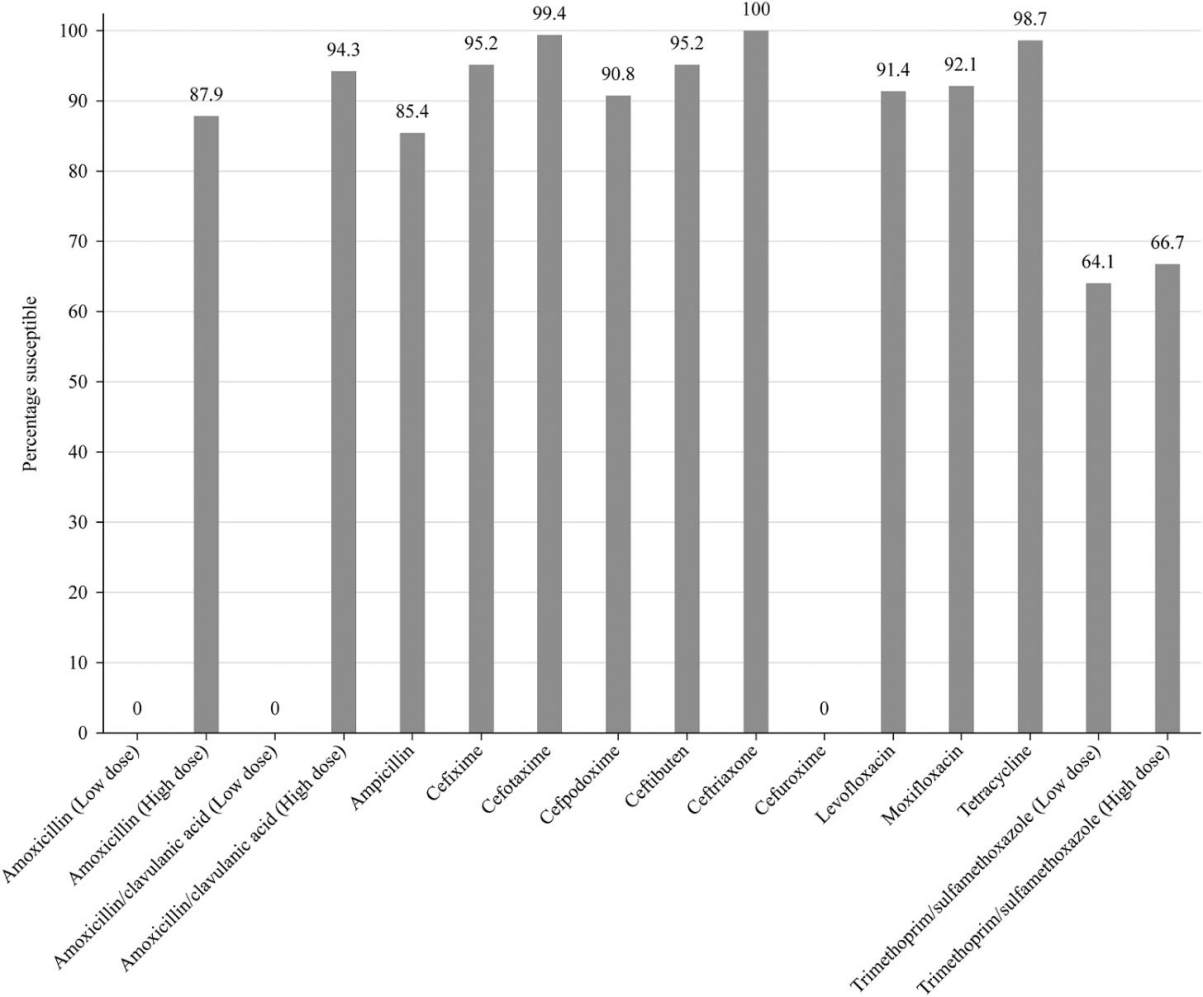
Antibiotic susceptibility rates of *H. influenzae* isolates (*n* = 315) from Türkiye based on EUCAST (dose-specific) breakpoints.

Summary MIC, susceptibility and MIC distribution data for all 315 *H. influenzae* isolates are given in Tables 7–9 and S2 and shown in Figures 5 and 6.

**Table 7.** MIC and susceptibility data for *H. influenzae* isolates (*n* = 315) from Türkiye using CLSI breakpoints

	MIC (mg/L)			CLSI susceptibility		
Antimicrobial	Range	50%	90%	%S	%I	%R
Amoxicillin	≤0.03–128	0.5	4	—	—	—
Ampicillin	≤0.03–128	0.25	2	85.4	6.0	8.6
Amoxicillin/clavulanic acid (2:1)	0.06–8	0.5	2	91.8	6.0	2.2
Cefaclor	≤0.25–32	2	8	93.3	5.7	1.0
Cefdinir	≤0.06–4	0.25	1	95.6	—	—
Cefixime	≤0.008–2	0.03	0.06	99.7	—	—
Cefotaxime	≤0.002–0.25	0.015	0.06	100	—	—
Cefpodoxime	≤0.015–2	0.06	0.25	100	—	—
Ceftibuten	≤0.008–>4	0.06	0.5	99.0	—	—
Ceftriaxone	≤0.001–0.12	0.004	0.015	100	—	—
Cefuroxime	≤0.03–16	0.5	2	99.4	0.3	0.3
Azithromycin	≤0.12–>8	1	1	99.1	—	—
Clarithromycin	≤0.25–>32	4	8	96.5	2.5	1.0
Tetracycline	≤0.12–16	0.25	0.5	98.7	1.0	0.3
Levofloxacin	≤0.004–>8	0.015	0.03	94.9	—	—
Moxifloxacin	≤0.004–>8	0.015	0.03	94.6	—	—
Trimethoprim/sulfamethoxazole	≤0.008–>8	0.12	8	64.1	6.0	29.8

—, not applicable; I, intermediate; R, resistant; S, susceptible.

**Table 8.** MIC and susceptibility data for *H. influenzae* isolates (*n* = 315) from Türkiye using EUCAST (dose-specific) breakpoints

	MIC (mg/L)			EUCAST susceptibility		
Antimicrobial	Range	50%	90%	%S	%I	%R
Amoxicillin (0.5 g × 3 oral)	≤0.03–128	0.5	4	0	87.9	12.1
Amoxicillin (0.75–1 g × 3 oral)	≤0.03–128	0.5	4	87.9	—	12.1
Ampicillin	≤0.03–128	0.25	2	85.4	—	14.6
Amoxicillin/clavulanic acid [2 mg/L] (0.5 g/0.125 g × 3 oral)	≤0.03–8	0.5	2	0	94.3	5.7
Amoxicillin/clavulanic acid [2 mg/L] (0.875 g/0.125 g × 3 oral)	≤0.03–8	0.5	2	94.3	—	5.7
Cefaclor	≤0.25–32	2	8	—	—	—
Cefdinir	≤0.06–4	0.25	1	—	—	—
Cefixime	≤0.008–2	0.03	0.06	95.2	—	4.8
Cefotaxime	≤0.002–0.25	0.015	0.06	99.4	—	0.6
Cefpodoxime	≤0.015–2	0.06	0.25	90.8	—	9.2
Ceftibuten	≤0.008–>4	0.06	0.5	95.2	—	4.8
Ceftriaxone	≤0.001–0.12	0.004	0.015	100	—	0
Cefuroxime	≤0.03–16	0.5	2	0	71.1	28.9
Azithromycin	≤0.12–>8	1	1	—	—	—
Clarithromycin	≤0.25–>32	4	8	—	—	—
Tetracycline	≤0.12–16	0.25	0.5	98.7	—	1.3
Levofloxacin	≤0.004–>8	0.015	0.03	91.4	—	8.6
Moxifloxacin	≤0.004–>8	0.015	0.03	92.1	—	7.9
Trimethoprim/sulfamethoxazole (0.16 g/0.8 g × 2 oral or IV)	≤0.008–>8	0.12	8	64.1	2.5	33.3
Trimethoprim/sulfamethoxazole (0.24 g/1.2 g × 2 oral or IV)	≤0.008–>8	0.12	8	66.7	—	33.3

—, not applicable; I, susceptible, increased exposure; R, resistant; S, susceptible.

**Table 9.** Summary MIC and susceptibility data for *H. influenzae* (*n* = 315) from Türkiye using PK/PD breakpoints

	MIC (mg/L)			PK/PD susceptibility
Antimicrobial	Range	50%	90%	%S
Amoxicillin (1.5 g/day)	≤0.03–128	0.5	4	87.9
Amoxicillin (4 g/day)	≤0.03–128	0.5	4	92.4
Amoxicillin/clavulanic acid (1.75 g/0.25 g/day adults; 45 mg/6.4 mg/kg/day children)	0.06–8	0.5	2	91.7
Amoxicillin/clavulanic acid (4 g/0.25 g/day adults; 90 mg/6.4 mg/kg/day children)	0.06–8	0.5	2	97.8
Ampicillin	≤0.03–128	0.25	2	—
Cefaclor	≤0.25–32	2	8	6.7
Cefdinir	≤0.06–4	0.25	1	56.5
Cefixime	≤0.008–2	0.03	0.06	99.7
Cefotaxime	≤0.002–0.25	0.015	0.06	—
Cefpodoxime	≤0.015–2	0.06	0.25	97.5
Ceftibuten	≤0.008–>4	0.06	0.5	—
Ceftriaxone	≤0.001–0.12	0.004	0.015	100
Cefuroxime	≤0.03–16	0.5	2	71.1
Azithromycin	≤0.12–>8	1	1	2.5
Clarithromycin	≤0.25–>32	4	8	1.0
Tetracycline	≤0.12–16	0.25	0.5	—
Levofloxacin	≤0.004–>8	0.015	0.03	94.9
Moxifloxacin	≤0.004–>8	0.015	0.03	94.6
Trimethoprim/sulfamethoxazole	≤0.008–>8	0.12	8	64.1

—, not applicable; PK/PD, pharmacokinetic/pharmacodynamic; S, susceptible.

### *H. influenzae* susceptibility

Most isolates of *H. influenzae* from Türkiye were β-lactamase-negative (294/315, 93.3%). Within this population, 26 isolates (8.3% of total *H. influenzae*) were β-lactamase negative ampicillin-resistant (BLNAR) by EUCAST breakpoints (ampicillin MIC ≥2 mg/L) and eight (2.5% of total *H. influenzae*) by CLSI breakpoints (ampicillin MIC ≥4 mg/L). One β-lactamase-positive isolate was found to be ampicillin susceptible (CLSI), which most likely is because the nitrocefin test is more sensitive than ampicillin MIC testing. In keeping with this β-lactamase and BLNAR status, 85.4% of isolates were susceptible to ampicillin (CLSI or EUCAST breakpoints). Amoxicillin breakpoints are not provided by CLSI, but EUCAST breakpoints at high dose indicated 87.9% susceptibility. At low dose, no isolate would be considered susceptible. High-dose PK/PD breakpoints for amoxicillin increased susceptibility to 92.4%. Susceptibility of isolates to amoxicillin/clavulanic acid was: 91.8% by CLSI breakpoints, 94.3% by EUCAST high-dose breakpoints and 97.8% by PK/PD high-dose breakpoints. Susceptibility was ≥93.3% for all of the cephalosporins according to CLSI breakpoints. Similar results were seen with EUCAST breakpoints (although these are not provided for cefaclor or cefdinir), except for cefuroxime (99.4% by CLSI versus 0% by EUCAST). Cefaclor, cefdinir and cefuroxime susceptibilities by PK/PD breakpoints were lower by CLSI (6.7%, 56.5% and 71.1%, respectively). Macrolide breakpoints were not provided by EUCAST against *H. influenzae*, but 99.1% susceptibility was observed for azithromycin and 96.5% susceptibility for clarithromycin by CLSI breakpoints. Macrolide susceptibility by PK/PD breakpoints was 2.5% and 1.0% for azithromycin and clarithromycin, respectively, in keeping with the lack of breakpoint provision for EUCAST. Both CLSI and EUCAST breakpoint standards showed 98.7% susceptibility to tetracycline, but PK/PD breakpoints are not given. All breakpoints showed 91.4%–94.9% fluoroquinolone susceptibility. Trimethoprim/sulfamethoxazole showed the weakest activity, with 64.1% susceptibility by CLSI, low-dose EUCAST and PK/PD breakpoints and 66.7% susceptibility by high-dose EUCAST breakpoints (Tables 7–9 and Figures 5 and 6).

### Comparative susceptibility of *H. influenzae* collected in 2015–17 and 2018–21

**Figure 7.**
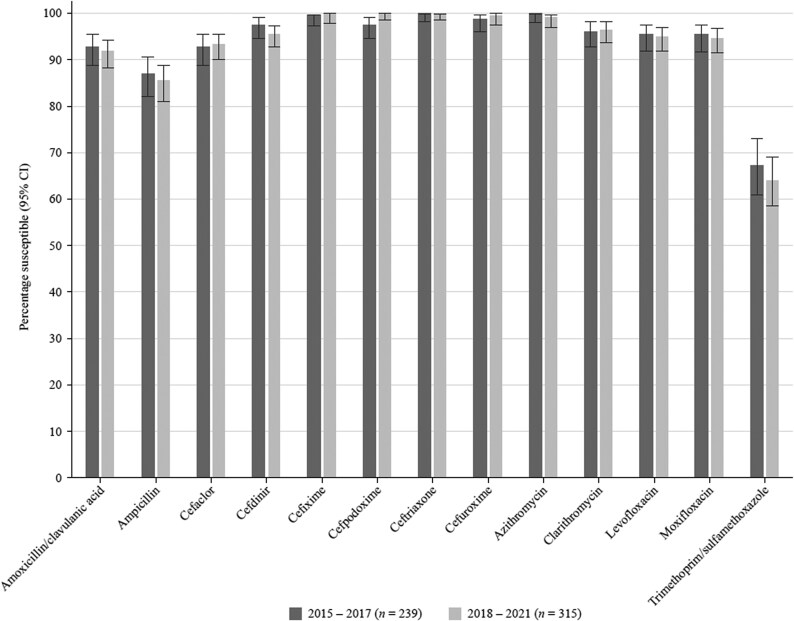
Comparison of antibiotic susceptibility rates of *H. influenzae* isolates from Türkiye collected in 2015–17 with isolates collected in 2018–21 (CLSI breakpoints). CI, confidence interval.

There was no significant change in susceptibility when comparing data from 2015 to 2017 with data from 2018 to 2021 (Figure 7).

## Discussion

SOAR is an ongoing global surveillance study focusing on the two main CA-RTIs pathogens, *S. pneumoniae* and *H. influenzae,* that has monitored numerous countries since 2002, including Türkiye. The data presented here are an analysis of the antibiotic susceptibility of *S. pneumoniae* and *H. influenzae* isolates collected in Türkiye from four centres between 2018 and 2021. This study may be limited in terms of national antibiotic resistance representation, given that samples were obtained from only four centres across two regions. However, the fact that most isolates were from presumably unrelated community-acquired infections suggests that the results are reflective of the broader Turkish community. Three of the four centres participated in the previous SOAR surveillance from 2015 to 2017 (Ege University, Hacettepe University Faculty of Medicine and Istanbul Faculty of Medicine, Istanbul University),^18^ and a direct statistical comparison between the two study periods is presented here. This continuity is important because inherent differences can occur from centre to centre within the same country, as observed in SOAR Türkiye 2011–13.^19^

The penicillin susceptibility results for *S. pneumoniae* in Türkiye 2018–21 confirm that oral penicillin or low-dose IV penicillin is not an appropriate treatment regimen for CA-RTIs, with EUCAST low-dose IV or CLSI oral breakpoints showing only 35.9% susceptibility. Although this is a numerical increase compared with SOAR 2015–17 (27.9% susceptible), it is not statistically significant. It is possible that susceptibility to oral penicillin has plateaued after the steep decline from SOAR 2002–09, when oral penicillin susceptibility was 74.7%.^20,21^ Both CLSI and EUCAST guidelines indicate that higher-dose IV penicillin is a better option, with susceptibility at 82.4%. CLSI and PK/PD breakpoints demonstrate a similar level of susceptibility for amoxicillin, amoxicillin/clavulanic acid, ceftriaxone and cefotaxime. EUCAST high-dose ceftriaxone susceptibility was 99.3%, but EUCAST breakpoints, even at the higher dose, indicate lower susceptibility for amoxicillin, amoxicillin/clavulanic acid and cefotaxime (≤62.7%) than CLSI. Although the distinction between CLSI and EUCAST is most likely based on different criteria used to determine breakpoints, this has important practical implications for clinical laboratories, especially in Türkiye, where it has been suggested that clinical laboratories are in transition from CLSI to EUCAST guidelines and breakpoints.^21^ Susceptibility according to both guidelines indicates poor activity for macrolides, tetracyclines and trimethoprim/sulfamethoxazole but good activity for fluoroquinolones against *S. pneumoniae* from Türkiye. Furthermore, apart from fluoroquinolones, there was a clear association between low penicillin susceptibility and low susceptibility to other antibiotics, as observed with the SOAR 2015–17 data.^18^ A recent report of surveillance data from 17 hospitals in Türkiye from 2016 to 2019 using EUCAST breakpoints indicated a similar level of low susceptibility to erythromycin and trimethoprim/sulfamethoxazole and high susceptibility to fluoroquinolones, as seen in the current study; however, a higher level of penicillin susceptibility (63.0%) was observed.^22^

In this study, we compared the susceptibility of pneumococci using CLSI breakpoints for isolates previously collected in 2015–17 from Türkiye with susceptibility from the current study (2018–21). As noted above for oral penicillin, there was no significant difference in susceptibility between the two study periods, except for cefaclor and trimethoprim/sulfamethoxazole, where susceptibility increased. Although a statistically significant increase in the percent susceptibility occurred for both agents, the percentage remained low. It is interesting to observe that a study from Izmir, Türkiye, also found a decrease in trimethoprim/sulfamethoxazole resistance from 51.6% in 2018 to 32.8% in 2019.^23^ The data from this study also suggest higher penicillin susceptibility, but this is not clear because susceptibility was inferred from oxacillin disc screening rather than MIC.^23^ This study also observed consistently higher tetracycline resistance each year from 2013 to 2019 than that observed in any SOAR study.^23^

*H. influenzae* isolates from Türkiye were predominantly β-lactamase negative (93.3%), with 26 BLNAR isolates using EUCAST breakpoints and eight by CLSI breakpoints. Apart from trimethoprim/sulfamethoxazole (64.1% susceptible), susceptibility to antibiotics was ≥85.4% by CLSI breakpoints. SOAR surveillance from 2002 to 2013 also indicated generally high antibiotic susceptibility with *H. influenzae*.^20,21^ No statistical difference in susceptibility by CLSI was observed between the 2015–18 and 2019–21 time periods. However, there are differences in susceptibility between CLSI, EUCAST and PK/PD breakpoints for cefuroxime (0% EUCAST, 71.7% PK/PD, 99.4% CLSI-susceptible in 2019–21) and macrolides (no EUCAST breakpoints given, 1.0%–2.5% PK/PD, 96.5%–99.1% CLSI). It is of interest to note that the high level of antibiotic susceptibility in *H. influenzae* contrasts greatly with the low level of antibiotic susceptibility observed with pneumococci. Data from the ATLAS study confirm high antimicrobial agent susceptibility in *H. influenzae* from Türkiye between 2015 and 2017 using CLSI breakpoints.^21^ However, the Guclu *et al.*^22^ study, mentioned above, found considerably higher ampicillin resistance (48.6%) and amoxicillin/clavulanic acid resistance (35.7%) in *H. influenzae* from Türkiye than those observed in the current study and the ATLAS study.^21^ The Guclu *et al.* (2021) study used older EUCAST breakpoints published in 2016; however, neither the ampicillin nor the amoxicillin/clavulanic acid breakpoint has changed over this time. Unfortunately, Guclu *et al.* (2021) did not report β-lactamase prevalence, which would have allowed for a useful comparison. Interestingly, cefuroxime and trimethoprim/sulfamethoxazole resistance was virtually identical between the current study and the Guclu *et al.* (2021) study. Tetracycline resistance in *H. influenzae* was higher in the Guclu *et al.* (2021) study than in the current study, but this can be explained by an increase in the EUCAST breakpoint for tetracycline against *H. influenzae* since 2016.

To conclude, antibiotic susceptibility in *S. pneumoniae* was generally <60% except for the fluoroquinolones (97.9%–99.3%), amoxicillin and amoxicillin/clavulanic acid (78.9%–79.6%, CLSI), cefotaxime and ceftriaxone (62.7%–82.4%). In contrast, *H. influenzae* susceptibility to most antibiotics was >90% except for trimethoprim/sulfamethoxazole (64.1%) and ampicillin (85.4%). Susceptibility of both organisms remained effectively unchanged since 2015. Continued surveillance of antibiotic susceptibility in Türkiye is required to regularly assess any future changes.

## Supplementary Material

dkaf289_Supplementary_Data

## Supplementary data

Tables S1 and S2 are available as Supplementary data at *JAC* Online.
