# Supplementary material for: Results from the Survey of Antibiotic Resistance (SOAR) 2018–21 in Türkiye: data based on CLSI, EUCAST (dose-specific) and pharmacokinetic/pharmacodynamic (PK/PD) breakpoints
Source: J Antimicrob Chemother. 2025 Nov 24;80(Suppl 3):iii98–iii113. doi: 10.1093/jac/dkaf289 (PMC12641130; doi:10.1093/jac/dkaf289)
Supplement: dkaf289_Supplementary_Data [file dkaf289_supplementary_data.docx]

**Results from the Survey of Antibiotic Resistance (SOAR) 2018 – 21 in Türkiye: data based on CLSI, EUCAST (dose-specific) and pharmacokinetic/pharmacodynamic (PK/PD) breakpoints**

**Authors:** Didem TORUMKUNEY^1^, Nergis KELES^2^, Ufuk HASDEMIR^3^, Gülşen HAZIROLAN^4^, Sohret AYDEMIR^5^, Zerrin AKTAS^6^, Oral ONCUL^7^, Ian MORRISSEY^8^, Anand MANOHARAN^9*^

**Affiliations:** ^1^GSK, London, UK; ^2^GSK, Istanbul, Türkiye; ^3^Department of Medical Microbiology, Marmara University School of Medicine, Pendik Training and Research Hospital, Istanbul, Türkiye; ^4^Department of Medical Microbiology, Hacettepe University Faculty of Medicine, Ankara, Türkiye; ^5^Department of Clinical Microbiology, Faculty of Medicine, Ege University, Izmir, Türkiye; ^6^Department of Clinical Microbiology, Istanbul Faculty of Medicine, Istanbul University, Istanbul, Türkiye; ^7^Department of Infectious Diseases and Clinical Microbiology, Istanbul Faculty of Medicine, Istanbul University, Istanbul, Türkiye; ^8^Antimicrobial Focus Ltd., Sawbridgeworth, UK; ^9^Infectious Diseases Medical & Scientific Affairs, GSK, Mumbai, India

*Corresponding author. E-mail: [anand.x.manoharan@gsk.com](mailto:anand.x.manoharan@gsk.com)

**Running title:** Survey of Antibiotic Resistance (SOAR) in Türkiye in 2018 – 21

**Supplementary Table 1.** MIC distribution data for *S. pneumoniae* isolates (*n* = 142) from Türkiye

|  |  | | Number of isolates at MIC (mg/L) | | | | | | | | | | | | | | | | | | | |
| --- | --- | --- | --- | --- | --- | --- | --- | --- | --- | --- | --- | --- | --- | --- | --- | --- | --- | --- | --- | --- | --- | --- |
| Antimicrobial | | | | ≤0.008 | ≤0.015 | 0.015 | ≤0.03 | 0.03 | ≤0.06 | 0.06 | 0.12 | ≤0.25 | 0.25 | 0.5 | 1 | 2 | 4 | >4 | 8 | >8 | 16 | >16 |
| AMX | | N | | 2 | – | 28 | – | 19 | – | 5 | 8 | – | 17 | 6 | 7 | 20 | 12 | – | 17 | 1 | – | – |
|  |  | Cum. % | | 1.4 | – | 21.1 | – | 34.5 | – | 38.0 | 43.7 | – | 55.6 | 59.9 | 64.8 | 78.9 | 87.3 | – | 99.3 | 100 | – | – |
|  |  | % | | 1.4 | – | 19.7 | – | 13.4 | – | 3.5 | 5.6 | – | 12.0 | 4.2 | 4.9 | 14.1 | 8.5 | – | 12.0 | 0.7 | – | – |
| AMC (2:1) | | N | | 1 | – | 20 | – | 30 | – | 3 | 9 | – | 13 | 7 | 9 | 21 | 12 | – | 16 | 1 | – | – |
|  |  | Cum. % | | 0.7 | – | 14.8 | – | 35.9 | – | 38.0 | 44.4 | – | 53.5 | 58.5 | 64.8 | 79.6 | 88.0 | – | 99.3 | 100 | – | – |
|  |  | % | | 0.7 | – | 14.1 | – | 21.1 | – | 2.1 | 6.3 | – | 9.2 | 4.9 | 6.3 | 14.8 | 8.5 | – | 11.3 | 0.7 | – | – |
| AMC  [2 mg/L] | | N | | – | – | 3 | – | 22 | – | 25 | 3 | – | 5 | 16 | 10 | – | 9 | – | 22 | 27 | – | – |
|  |  | Cum. % | | – | – | 2.1 | – | 17.6 | – | 35.2 | 37.3 | – | 40.8 | 52.1 | 59.2 | – | 65.5 | – | 81.0 | 100 | – | – |
|  |  | % | | – | – | 2.1 | – | 15.5 | – | 17.6 | 2.1 | – | 3.5 | 11.3 | 7.0 | – | 6.3 | – | 15.5 | 19.0 | – | – |
| AZM | | N | | – | 2 | – | – | 17 | – | 58 | – | – | 1 | – | 1 | 5 | 7 | – | 3 | – | 1 | 47 |
|  |  | Cum. % | | – | 1.4 | – | – | 13.4 | – | 54.2 | – | – | 54.9 | – | 55.6 | 59.2 | 64.1 | – | 66.2 | – | 66.9 | 100 |
|  |  | % | | – | 1.4 | – | – | 12.0 | – | 40.8 | – | – | 0.7 | – | 0.7 | 3.5 | 4.9 | – | 2.1 | – | 0.7 | 33.1 |
| CEC | | N | | – | – | – | – | – | – | – | – | – | 7 | 42 | 11 | 8 | 5 | 69 | – | – | – | – |
|  |  | Cum. % | | – | – | – | – | – | – | – | – | – | 4.9 | 34.5 | 42.3 | 47.9 | 51.4 | 100 | – | – | – | – |
|  |  | % | | – | – | – | – | – | – | – | – | – | 4.9 | 29.6 | 7.7 | 5.6 | 3.5 | 48.6 | – | – | – | – |
| CDR | | N | | – | – | – | – | 5 | – | 41 | 8 | – | 8 | 17 | 5 | 6 | 12 | – | 19 | 21 | – | – |
|  |  | Cum. % | | – | – | – | – | 3.5 | – | 32.4 | 38.0 | – | 43.7 | 55.6 | 59.2 | 63.4 | 71.8 | – | 85.2 | 100 | – | – |
|  |  | % | | – | – | – | – | 3.5 | – | 28.9 | 5.6 | – | 5.6 | 12.0 | 3.5 | 4.2 | 8.5 | – | 13.4 | 14.8 | – | – |
| CFM | | N | | – | – | – | – | – | – | – | – | 45 | – | 3 | 13 | 14 | 7 | – | 7 | – | 17 | 36 |
|  |  | Cum. % | | – | – | – | – | – | – | – | – | 31.7 | – | 33.8 | 43.0 | 52.8 | 57.7 | – | 62.7 | – | 74.6 | 100 |
|  |  | % | | – | – | – | – | – | – | – | – | 31.7 | – | 2.1 | 9.2 | 9.9 | 4.9 | – | 4.9 | – | 12.0 | 25.4 |
| CTX | | N | | 4 | – | 31 | – | 12 | – | 7 | 22 | – | 6 | 7 | 22 | 27 | 4 | – | – | – | – | – |
|  |  | Cum. % | | 2.8 | – | 24.6 | – | 33.1 | – | 38.0 | 53.5 | – | 57.7 | 62.7 | 78.2 | 97.2 | 100 | – | – | – | – | – |
|  |  | % | | 2.8 | – | 21.8 | – | 8.5 | – | 4.9 | 15.5 | – | 4.2 | 4.9 | 15.5 | 19.0 | 2.8 | – | – | – | – | – |
| CPD | | N | | – | 11 | – | – | 35 | – | 2 | 11 | – | 18 | 6 | 7 | 13 | 23 | 16 | – | – | – | – |
|  |  | Cum. % | | – | 7.7 | – | – | 32.4 | – | 33.8 | 41.5 | – | 54.2 | 58.5 | 63.4 | 72.5 | 88.7 | 100 | – | – | – | – |
|  |  | % | | – | 7.7 | – | – | 24.6 | – | 1.4 | 7.7 | – | 12.7 | 4.2 | 4.9 | 9.2 | 16.2 | 11.3 | – | – | – | – |
| CTB | | N | | – | – | – | – | – | – | – | – | – | – | – | – | 10 | 37 | – | 5 | – | 9 | 81 |
|  |  | Cum. % | | – | – | – | – | – | – | – | – | – | – | – | – | 7.0 | 33.1 | – | 36.6 | – | 43.0 | 100 |
|  |  | % | | – | – | – | – | – | – | – | – | – | – | – | – | 7.0 | 26.1 | – | 3.5 | – | 6.3 | 57.0 |
| CRO | | N | | 2 | – | 29 | – | 16 | – | 6 | 17 | – | 10 | 9 | 28 | 24 | 1 | – | – | – | – | – |
|  |  | Cum. % | | 1.4 | – | 21.8 | – | 33.1 | – | 37.3 | 49.3 | – | 56.3 | 62.7 | 82.4 | 99.3 | 100 | – | – | – | – | – |
|  |  | % | | 1.4 | – | 20.4 | – | 11.3 | – | 4.2 | 12.0 | – | 7.0 | 6.3 | 19.7 | 16.9 | 0.7 | – | – | – | – | – |
| CXM | | N | | 1 | – | 17 | – | 27 | – | 1 | 6 | – | 13 | 16 | 3 | 7 | 22 | – | 8 | 21 | – | – |
|  |  | Cum. % | | 0.7 | – | 12.7 | – | 31.7 | – | 32.4 | 36.6 | – | 45.8 | 57.0 | 59.2 | 64.1 | 79.6 | – | 85.2 | 100 | – | – |
|  |  | % | | 0.7 | – | 12.0 | – | 19.0 | – | 0.7 | 4.2 | – | 9.2 | 11.3 | 2.1 | 4.9 | 15.5 | – | 5.6 | 14.8 | – | – |
| CLR | | N | | – | 52 | – | – | 25 | – | – | – | – | 1 | 2 | 11 | 1 | 2 | – | 2 | – | – | 46 |
|  |  | Cum. % | | – | 36.6 | – | – | 54.2 | – | – | – | – | 54.9 | 56.3 | 64.1 | 64.8 | 66.2 | – | 67.6 | – | – | 100 |
|  |  | % | | – | 36.6 | – | – | 17.6 | – | – | – | – | 0.7 | 1.4 | 7.7 | 0.7 | 1.4 | – | 1.4 | – | – | 32.4 |
| DOX | | N | | – | – | – | – | 7 | – | 65 | 11 | – | – | 1 | 1 | 6 | 26 | 25 | – | – | – | – |
|  |  | Cum. % | | – | – | – | – | 4.9 | – | 50.7 | 58.5 | – | – | 59.2 | 59.9 | 64.1 | 82.4 | 100 | – | – | – | – |
|  |  | % | | – | – | – | – | 4.9 | – | 45.8 | 7.7 | – | – | 0.7 | 0.7 | 4.2 | 18.3 | 17.6 | – | – | – | – |
| ERY | | N | | – | 12 | – | – | 65 | – | – | – | – | – | 1 | 2 | 10 | 3 | – | 2 | – | 1 | 46 |
|  |  | Cum. % | | – | 8.5 | – | – | 54.2 | – | – | – | – | – | 54.9 | 56.3 | 63.4 | 65.5 | – | 66.9 | – | 67.6 | 100 |
|  |  | % | | – | 8.5 | – | – | 45.8 | – | – | – | – | – | 0.7 | 1.4 | 7.0 | 2.1 | – | 1.4 | – | 0.7 | 32.4 |
| LVX | | N | | – | – | – | – | – | – | – | – | – | – | 32 | 97 | 10 | 2 | – | – | 1 | – | – |
|  |  | Cum. % | | – | – | – | – | – | – | – | – | – | – | 22.5 | 90.8 | 97.9 | 99.3 | – | – | 100 | – | – |
|  |  | % | | – | – | – | – | – | – | – | – | – | – | 22.5 | 68.3 | 7.0 | 1.4 | – | – | 0.7 | – | – |
| MXF | | N | | – | – | – | 3 | – | – | 63 | 74 | – | 1 | – | – | – | 1 | – | – | – | – | – |
|  |  | Cum. % | | – | – | – | 2.1 | – | – | 46.5 | 98.6 | – | 99.3 | – | – | – | 100 | – | – | – | – | – |
|  |  | % | | – | – | – | 2.1 | – | – | 44.4 | 52.1 | – | 0.7 | – | – | – | 0.7 | – | – | – | – | – |
| PEN | | N | | 3 | – | 39 | – | 6 | – | 3 | 10 | – | 20 | 4 | 12 | 20 | 24 | – | 1 | – | – | – |
|  |  | Cum. % | | 2.1 | – | 29.6 | – | 33.8 | – | 35.9 | 43.0 | – | 57.0 | 59.9 | 68.3 | 82.4 | 99.3 | – | 100 | – | – | – |
|  |  | % | | 2.1 | – | 27.5 | – | 4.2 | – | 2.1 | 7.0 | – | 14.1 | 2.8 | 8.5 | 14.1 | 16.9 | – | 0.7 | – | – | – |
| TET | | N | | – | – | – | – | – | – | 2 | 54 | – | 23 | 2 | 2 | 1 | – | 58 | – | – | – | – |
|  |  | Cum. % | | – | – | – | – | – | – | 1.4 | 39.4 | – | 55.6 | 57.0 | 58.5 | 59.2 | – | 100 | – | – | – | – |
|  |  | % | | – | – | – | – | – | – | 1.4 | 38.0 | – | 16.2 | 1.4 | 1.4 | 0.7 | – | 40.8 | – | – | – | – |
| SXT | | N | | – | – | – | – | – | 1 | – | 14 | – | 55 | 5 | 11 | 5 | 14 | – | 17 | 20 | – | – |
|  |  | Cum. % | | – | – | – | – | – | 0.7 | – | 10.6 | – | 49.3 | 52.8 | 60.6 | 64.1 | 73.9 | – | 85.9 | 100 | – | – |
|  |  | % | | – | – | – | – | – | 0.7 | – | 9.9 | – | 38.7 | 3.5 | 7.7 | 3.5 | 9.9 | – | 12.0 | 14.1 | – | – |

–, not applicable; AMC, amoxicillin/clavulanic acid; AMX, amoxicillin; AZM, azithromycin; CDR, cefdinir; CEC, cefaclor; CFM, cefixime; CLR, clarithromycin; CPD, cefpodoxime; CRO, ceftriaxone; CTB, ceftibuten; CTX, cefotaxime; Cum., cumulative; CXM, cefuroxime; DOX, doxycycline; ERY, erythromycin; LVX, levofloxacin; MXF, moxifloxacin; PEN, penicillin; SXT, trimethoprim/sulfamethoxazole; TET, tetracycline.

Bold vertical bars in table correspond to the CLSI-susceptible breakpoints.

**Supplementary Table 2.** MIC distribution data for *H. influenzae* isolates (*n* = 315) from Türkiye

|  | | Number of isolates at MIC (mg/L) | | | | | | | | | | | | | | | | | | | | | | | | | | | | |
| --- | --- | --- | --- | --- | --- | --- | --- | --- | --- | --- | --- | --- | --- | --- | --- | --- | --- | --- | --- | --- | --- | --- | --- | --- | --- | --- | --- | --- | --- | --- |
| Antimicrobial | | ≤0.001 | ≤0.002 | 0.002 | ≤0.004 | 0.004 | ≤0.008 | 0.008 | ≤0.015 | 0.015 | ≤0.03 | 0.03 | ≤0.06 | 0.06 | ≤0.12 | 0.12 | ≤0.25 | 0.25 | 0.5 | 1 | 2 | 4 | >4 | 8 | >8 | 16 | 32 | >32 | 64 | 128 |
| AMX | N | – | – | – | – | – | – | – | – | – | 2 | – | – | – | – | 9 | – | 79 | 80 | 66 | 41 | 14 | – | 7 | – | 4 | 5 | – | 6 | 2 |
|  | Cum. % | – | – | – | – | – | – | – | – | – | 0.6 | – | – | – | – | 3.5 | – | 28.6 | 54.0 | 74.9 | 87.9 | 92.4 | – | 94.6 | – | 95.9 | 97.5 | – | 99.4 | 100 |
|  | % | – | – | – | – | – | – | – | – | – | 0.6 | – | – | – | – | 2.9 | – | 25.1 | 25.4 | 21.0 | 13.0 | 4.4 | – | 2.2 | – | 1.3 | 1.6 | – | 1.9 | 0.6 |
| AMC (2:1) | N | – | – | – | – | – | – | – | – | – | – | – | – | 1 | – | 7 | – | 58 | 99 | 77 | 47 | 19 | – | 7 | – | – | – | – | – | – |
|  | Cum. % | – | – | – | – | – | – | – | – | – | – | – | – | 0.3 | – | 2.5 | – | 21.0 | 52.4 | 76.8 | 91.7 | 97.8 | – | 100 | – | – | – | – | – | – |
|  | % | – | – | – | – | – | – | – | – | – | – | – | – | 0.3 | – | 2.2 | – | 18.4 | 31.4 | 24.4 | 14.9 | 6.0 | – | 2.2 | – | – | – | – | – | – |
| AMC  [2 mg/L] | N | – | – | – | – | – | – | – | – | – | 5 | – | – | 2 | – | 17 | – | 96 | 77 | 71 | 29 | 14 | – | 4 | – | – | – | – | – | – |
|  | Cum. % | – | – | – | – | – | – | – | – | – | 1.6 | – | – | 2.2 | – | 7.6 | – | 38.1 | 62.5 | 85.1 | 94.3 | 98.7 | – | 100 | – | – | – | – | – | – |
|  | % | – | – | – | – | – | – | – | – | – | 1.6 | – | – | 0.6 | – | 5.4 | – | 30.5 | 24.4 | 22.5 | 9.2 | 4.4 | – | 1.3 | – | – | – | – | – | – |
| AMP | N | – | – | – | – | – | – | – | – | – | 4 | – | – | 8 | – | 77 | – | 71 | 47 | 62 | 19 | 9 | – | – | – | 4 | 5 | – | 7 | 2 |
|  | Cum. % | – | – | – | – | – | – | – | – | – | 1.3 | – | – | 3.8 | – | 28.3 | – | 50.8 | 65.7 | 85.4 | 91.4 | 94.3 | – | – | – | 95.6 | 97.1 | – | 99.4 | 100 |
|  | % | – | – | – | – | – | – | – | – | – | 1.3 | – | – | 2.5 | – | 24.4 | – | 22.5 | 14.9 | 19.7 | 6.0 | 2.9 | – | – | – | 1.3 | 1.6 | – | 2.2 | 0.6 |
| AZM | N | – | – | – | – | – | – | – | – | – | – | – | – | – | 8 | – | – | 32 | 104 | 143 | 21 | 4 | – | – | 3 | – | – | – | – | – |
|  | Cum. % | – | – | – | – | – | – | – | – | – | – | – | – | – | 2.5 | – | – | 12.7 | 45.7 | 91.1 | 97.8 | 99.0 | – | – | 100 | – | – | – | – | – |
|  | % | – | – | – | – | – | – | – | – | – | – | – | – | – | 2.5 | – | – | 10.2 | 33.0 | 45.4 | 6.7 | 1.3 | – | – | 1.0 | – | – | – | – | – |
| CEC | N | – | – | – | – | – | – | – | – | – | – | – | – | – | – | – | 4 | – | 17 | 67 | 72 | 71 | – | 63 | – | 18 | 3 | – | – | – |
|  | Cum. % | – | – | – | – | – | – | – | – | – | – | – | – | – | – | – | 1.3 | – | 6.7 | 27.9 | 50.8 | 73.3 | – | 93.3 | – | 99.0 | 100 | – | – | – |
|  | % | – | – | – | – | – | – | – | – | – | – | – | – | – | – | – | 1.3 | – | 5.4 | 21.3 | 22.9 | 22.5 | – | 20.0 | – | 5.7 | 1.0 | – | – | – |
| CDR | N | – | – | – | – | – | – | – | – | – | – | – | 11 | – | – | 47 | – | 120 | 84 | 39 | 12 | 2 | – | – | – | – | – | – | – | – |
|  | Cum. % | – | – | – | – | – | – | – | – | – | – | – | 3.5 | – | – | 18.4 | – | 56.5 | 83.2 | 95.6 | 99.4 | 100 | – | – | – | – | – | – | – | – |
|  | % | – | – | – | – | – | – | – | – | – | – | – | 3.5 | – | – | 14.9 | – | 38.1 | 26.7 | 12.4 | 3.8 | 0.6 | – | – | – | – | – | – | – | – |
| CFM | N | – | – | – | – | – | 18 | – | – | 64 | – | 163 | – | 50 | – | 5 | – | 4 | 8 | 2 | 1 | – | – | – | – | – | – | – | – | – |
|  | Cum. % | – | – | – | – | – | 5.7 | – | – | 26.0 | – | 77.8 | – | 93.7 | – | 95.2 | – | 96.5 | 99.0 | 99.7 | 100 | – | – | – | – | – | – | – | – | – |
|  | % | – | – | – | – | – | 5.7 | – | – | 20.3 | – | 51.7 | – | 15.9 | – | 1.6 | – | 1.3 | 2.5 | 0.6 | 0.3 | – | – | – | – | – | – | – | – | – |
| CTX | N | – | 38 | – | – | 33 | – | 59 | – | 81 | – | 69 | – | 30 | – | 3 | – | 2 | – | – | – | – | – | – | – | – | – | – | – | – |
|  | Cum. % | – | 12.1 | – | – | 22.5 | – | 41.3 | – | 67.0 | – | 88.9 | – | 98.4 | – | 99.4 | – | 100 | – | – | – | – | – | – | – | – | – | – | – | – |
|  | % | – | 12.1 | – | – | 10.5 | – | 18.7 | – | 25.7 | – | 21.9 | – | 9.5 | – | 1.0 | – | 0.6 | – | – | – | – | – | – | – | – | – | – | – | – |
| CPD | N | – | – | – | – | – | – | – | 17 | – | – | 81 | – | 88 | – | 55 | – | 45 | 21 | 5 | 3 | – | – | – | – | – | – | – | – | – |
|  | Cum. % | – | – | – | – | – | – | – | 5.4 | – | – | 31.1 | – | 59.0 | – | 76.5 | – | 90.8 | 97.5 | 99.0 | 100 | – | – | – | – | – | – | – | – | – |
|  | % | – | – | – | – | – | – | – | 5.4 | – | – | 25.7 | – | 27.9 | – | 17.5 | – | 14.3 | 6.7 | 1.6 | 1.0 | – | – | – | – | – | – | – | – | – |
| CTB | N | – | – | – | – | – | 2 | – | – | 7 | – | 62 | – | 99 | – | 62 | – | 47 | 17 | 4 | 12 | 2 | 1 | – | – | – | – | – | – | – |
|  | Cum. % | – | – | – | – | – | 0.6 | – | – | 2.9 | – | 22.5 | – | 54.0 | – | 73.7 | – | 88.6 | 94.0 | 95.2 | 99.0 | 99.7 | 100 | – | – | – | – | – | – | – |
|  | % | – | – | – | – | – | 0.6 | – | – | 2.2 | – | 19.7 | – | 31.4 | – | 19.7 | – | 14.9 | 5.4 | 1.3 | 3.8 | 0.6 | 0.3 | – | – | – | – | – | – | – |
| CRO | N | 18 | – | 59 | – | 114 | – | 62 | – | 46 | – | 12 | – | 3 | – | 1 | – | – | – | – | – | – | – | – | – | – | – | – | – | – |
|  | Cum. % | 5.7 | – | 24.4 | – | 60.6 | – | 80.3 | – | 94.9 | – | 98.7 | – | 99.7 | – | 100 | – | – | – | – | – | – | – | – | – | – | – | – | – | – |
|  | % | 5.7 | – | 18.7 | – | 36.2 | – | 19.7 | – | 14.6 | – | 3.8 | – | 1.0 | – | 0.3 | – | – | – | – | – | – | – | – | – | – | – | – | – | – |
| CXM | N | – | – | – | – | – | – | – | – | – | 7 | – | – | 2 | – | 9 | – | 40 | 110 | 56 | 75 | 14 | – | 1 | – | 1 | – | – | – | – |
|  | Cum. % | – | – | – | – | – | – | – | – | – | 2.2 | – | – | 2.9 | – | 5.7 | – | 18.4 | 53.3 | 71.1 | 94.9 | 99.4 | – | 99.7 | – | 100 | – | – | – | – |
|  | % | – | – | – | – | – | – | – | – | – | 2.2 | – | – | 0.6 | – | 2.9 | – | 12.7 | 34.9 | 17.8 | 23.8 | 4.4 | – | 0.3 | – | 0.3 | – | – | – | – |
| CLR | N | – | – | – | – | – | – | – | – | – | – | – | – | – | – | – | 3 | – | 7 | 2 | 34 | 161 | – | 97 | – | 8 | 1 | 2 | – | – |
|  | Cum. % | – | – | – | – | – | – | – | – | – | – | – | – | – | – | – | 1.0 | – | 3.2 | 3.8 | 14.6 | 65.7 | – | 96.5 | – | 99.0 | 99.4 | 100 | – | – |
|  | % | – | – | – | – | – | – | – | – | – | – | – | – | – | – | – | 1.0 | – | 2.2 | 0.6 | 10.8 | 51.1 | – | 30.8 | – | 2.5 | 0.3 | 0.6 | – | – |
| LVX | N | – | – | – | 12 | – | – | 46 | – | 210 | – | 20 | – | – | – | 2 | – | – | 5 | 2 | 2 | – | – | 4 | 12 | – | – | – | – | – |
|  | Cum. % | – | – | – | 3.8 | – | – | 18.4 | – | 85.1 | – | 91.4 | – | – | – | 92.1 | – | – | 93.7 | 94.3 | 94.9 | – | – | 96.2 | 100 | – | – | – | – | – |
|  | % | – | – | – | 3.8 | – | – | 14.6 | – | 66.7 | – | 6.3 | – | – | – | 0.6 | – | – | 1.6 | 0.6 | 0.6 | – | – | 1.3 | 3.8 | – | – | – | – | – |
| MXF | N | – | – | – | 19 | – | – | 47 | – | 167 | – | 54 | – | 1 | – | 2 | – | – | 7 | 1 | – | 1 | – | 8 | 8 | – | – | – | – | – |
|  | Cum. % | – | – | – | 6.0 | – | – | 21.0 | – | 74.0 | – | 91.1 | – | 91.4 | – | 92.1 | – | – | 94.3 | 94.6 | – | 94.9 | – | 97.5 | 100 | – | – | – | – | – |
|  | % | – | – | – | 6.0 | – | – | 14.9 | – | 53.0 | – | 17.1 | – | 0.3 | – | 0.6 | – | – | 2.2 | 0.3 | – | 0.3 | – | 2.5 | 2.5 | – | – | – | – | – |
| TET | N | – | – | – | – | – | – | – | – | – | – | – | – | – | 5 | – | – | 202 | 104 | – | – | 3 | – | – | – | 1 | – | – | – | – |
|  | Cum. % | – | – | – | – | – | – | – | – | – | – | – | – | – | 1.6 | – | – | 65.7 | 98.7 | – | – | 99.7 | – | – | – | 100 | – | – | – | – |
|  | % | – | – | – | – | – | – | – | – | – | – | – | – | – | 1.6 | – | – | 64.1 | 33.0 | – | – | 1.0 | – | – | – | 0.3 | – | – | – | – |
| SXT | N | – | – | – | – | – | 4 | – | – | 7 | – | 31 | – | 75 | – | 68 | – | 12 | 5 | 8 | 11 | 29 | – | 49 | 16 | – | – | – | – | – |
|  | Cum. % | – | – | – | – | – | 1.3 | – | – | 3.5 | – | 13.3 | – | 37.1 | – | 58.7 | – | 62.5 | 64.1 | 66.7 | 70.2 | 79.4 | – | 94.9 | 100 | – | – | – | – | – |
|  | % | – | – | – | – | – | 1.3 | – | – | 2.2 | – | 9.8 | – | 23.8 | – | 21.6 | – | 3.8 | 1.6 | 2.5 | 3.5 | 9.2 | – | 15.6 | 5.1 | – | – | – | – | – |

–, not applicable; AMC, amoxicillin/clavulanic acid; AMP, ampicillin; AMX, amoxicillin; AZM, azithromycin; CDR, cefdinir; CEC, cefaclor; CFM, cefixime; CLR, clarithromycin; CPD, cefpodoxime; CRO, ceftriaxone; CTB, ceftibuten; CTX, cefotaxime; Cum., cumulative; CXM, cefuroxime; LVX, levofloxacin; MXF, moxifloxacin; SXT, trimethoprim/sulfamethoxazole; TET, tetracycline.

Bold vertical bars in table correspond to the CLSI-susceptible breakpoints.
